# Pirogow's Amputation: A Modification of the Operation Method

**DOI:** 10.1155/2013/460792

**Published:** 2013-03-28

**Authors:** M. Bueschges, T. Muehlberger, K. L. Mauss, J. C. Bruck, C. Ottomann

**Affiliations:** ^1^Sektion für Plastische Chirurgie und Handchirurgie, Intensiveinheit für Schwerbrandverletzte, Universitätsklinikum Schleswig Holstein Campus Lübeck, Ratzeburger Allee 160, 23560 Lübeck, Germany; ^2^Abteilung für Plastische Chirurgie, DRK Kliniken Berlin, Berlin, Germany; ^3^Abteilung für Plastische Chirurgie, Martin Luther Krankenhaus, Caspar-Theyß-Straße 27-31, Grunewald, 14193 Berlin, Germany

## Abstract

*Introduction*. Pirogow's amputation at the ankle presents a valuable alternative to lower leg amputation for patients with the corresponding indications. Although this method offers the ability to stay mobile without the use of a prosthesis, it is rarely performed. This paper proposes a modification regarding the operation method of the Pirogow amputation. The results of the modified operation method on ten patients were objectified 12 months after the operation using a patient questionnaire (Ankle Score). *Material and Methods*. We modified the original method by rotating the calcaneus. To fix the calcaneus to the tibia, Kirschner wire and a 3/0 spongiosa tension screw as well as a Fixateur externe were used. *Results*. 70% of those questioned who were amputated following the modified Pirogow method indicated an excellent or very good result in total points whereas in the control group (original Pirogow's amputation) only 40% reported excellent or very good result. In addition, the level of pain experienced one year after the completed operation showed different results in favour of the group being operated with the modified way. Furthermore, patients in both groups showed differences in radiological results, postoperative leg length difference, and postoperative mobility. *Conclusion*. The modified Pirogow amputation presents a valuable alternative to the original amputation method for patients with the corresponding indications. The benefits are found in the significantly reduced pain, difference in reduced radiological complications, the increase in mobility without a prosthesis, and the reduction of postoperative leg length difference.

## 1. Introduction

**Figure 1:**
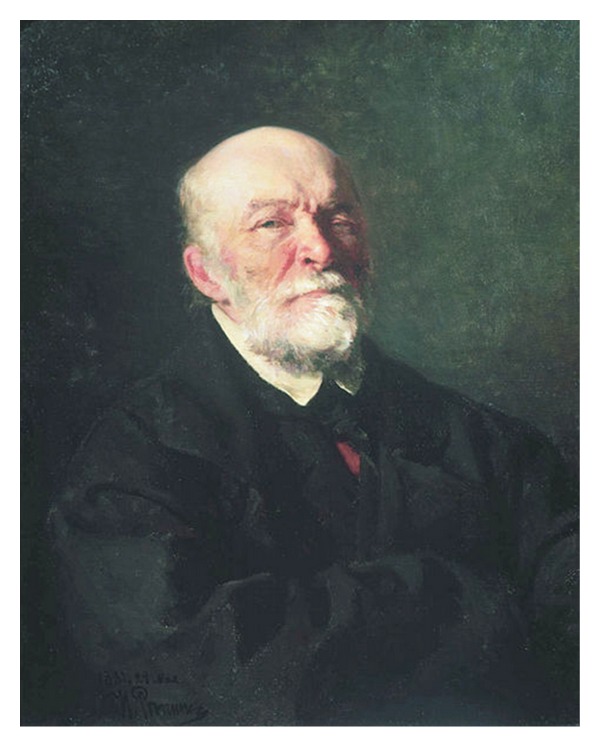
Nikolai Iwanowitsch Pirogow painted by Ilja Jefimowitsch Repin, 1881.

**Figure 2:**
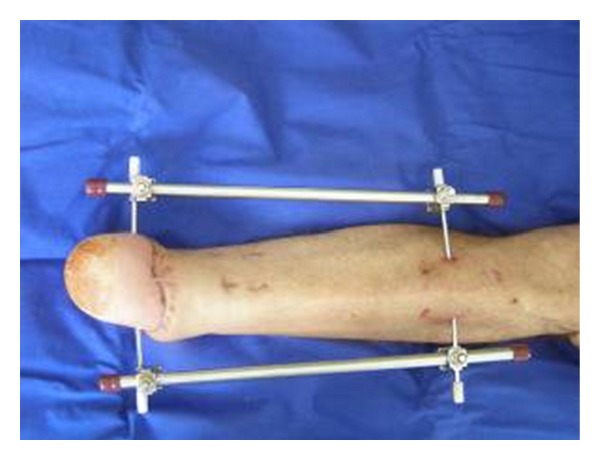
Temporary fixation by fixateur externe.

In his original article from 1854, Nikolai Iwanowitsch Pirogow reported on hundreds of lower limb amputations he carried out during the Crimean war [1] (Figure 1). The Crimean war was fought between Imperial Russia on one side and an alliance of France, the United Kingdom, the Kingdom of Sardinia, and the Ottoman Empire on the other. The goal of the anti-Russian alliance was to break the Russian position of power around the Black Sea and to put a halt to Russian expansion into the Balkan territory of the Ottoman Empire. The war broke out in 1853 on the Crimean peninsula and ended in 1856 with Russia's loss. The battles fought were not the only cause of the numerous lives lost during the war. Medical care during the war was incredibly substandard, causing the British nurse Florence Nightingale to construct and reorganize numerous military hospitals and care stations. The Crimean war saw an increased use of mines, and injuries to the foot and leg were becoming a more common occurrence. Due to this fact, Pirogow independently searched for a way to avoid an amputation of the limb below the knee [2]. He developed a method of amputation at the level of the ankle, which offers the patient a variety of benefits compared to the previously used transtibial amputation [3]. This method of amputation at the ankle reduced the mortality rate, which at that time laid between 25% and 50% of patients undergoing lower leg amputation [4]. A further important benefit was the significantly decreased difference in leg length, which allowed the patient to be mobile without the use of a prosthesis [5]. Due to the low level of prosthetic care at the time, this significantly reduced the patient's level of disability. In the scope of a Pirogow amputation, the average difference in leg length in comparison to the healthy extremity amounts to 2.8 cm [6]. Due to the fact that the original literature from Pirogow was not written in English, this method spread very slowly [7]. Even today this method is only rarely performed. A publication by AOK Germany from 2003 indicated that out of a total of 44,000 amputations on lower extremities performed, an amputation based on the Pirogow method was only carried out 44 times, which amounts to only 0.1% [8]. In contrast, a lower leg amputation was performed 8321 times in 2003, which amounts to 18% [8]. The authors claim that the original method of Pirogow's amputation can be advanced by rotating and fixing the calcaneus and by obtaining the operation goal by fixing the bones with a Fixateur externe (Figure 2).

Whether these modifications in Pirogow amputation were truly beneficial, ten patients were investigated postoperatively using a questionnaire. These results were compared to a control group of ten patients who were amputated using the original method.

## 2. Indications and Contraindications of the Pirogow Method of Amputation

The indications and contraindications of the Pirogow method of amputation are listed in Table 1.

### 2.1. Surgical Technique

It is recommended to perform an angiography on patients to show the supply of blood to the lower extremities in the heel area when an intact perfusion of the tibialis posterior is doubted. The goal of the amputation technique presented is the complete preservation of the plantar fascia and a large part of the calcaneus in order to preserve the full length of the leg, the mechanically stable skin of the heel with preservation of the original sensation, and the subcutaneous tissue in this area as a contact surface that is well cushioned against shear force and walking processes [9]. Disruptions to wound healing during the postoperative phase are mostly attributable to disrupted circulation in the plantar fascia, and a failure of the calcaneus to fuse with the tibia, which is why a nontraumatic operational technique protecting the tibialis posterior as much as possible, can be seen as a prerequisite [10]. A modified fish-mouth incision is drawn. Two points 1 cm plantar and ventral to the lateral and medial malleolus serve as key points of the incision. These points are joined plantarly and dorsally and result in the edges of the excision, angled to the thigh at 90°. It is recommended to leave at least 6 cm soft tissue of the sole to allow for the wound edges to be reduced as needed during the course of the operation. One begins with the incision at the dorsal marking between the key points along the fascia. The tendons of the foot extensor including the m. tibialis anterior are shortened. The a. dorsalis pedis or the a. tibialis anterior, if necessary, are ligated during the course of the procedure. After the plantar incision is made, the n. suralis and the n. tibialis should be cut through as proximally as possible. Afterward the plantar fascia as well as the foot flexors should be cut while under traction. Afterwards, the midtarsal joint is disarticulated [11]. In order to remove the talus bone, the anterior talocalcaneal ligament deep in the sinus tarsi must be cut through. Now, we modify the original method through anchoring the calcaneus by horizontally drilling a Steinmann nail 2 cm cranial and anterior to the calcaneal spur to allow the use of an external fixator. Additionally, a further Steinmann nail is drilled horizontally approx. 20 cm proximal through the front edge of the tibia. The calcaneus together with the plantar heel lobe is then turned at a 60–90 degree angle to correspond with the associated distal tibia surface, whereas Pirogow did not rotate the calcaneus. In order to achieve an adequate degree of rotation, it is advantageous to incise the Achilles tendon or cut it through completely [12]. As a further modification to ensure a stable bone jointment of tibia and calcaneus, a fixateur externe is used (Figure 2) and, after temporarily fixating the calcaneus to the tibia, osteosynthesis is carried out using a Kirschner wire and a 3/0 spongiosa tension screw. After ample haemostasis, the wound can be closed using strong and deep backstitch sutures upon inserting Redon drains.

### 2.2. Prosthetic Care after Pirogow Amputation

An interim prosthesis can be fitted after the fourth postoperative week. This enables the patient to become mobile with slight pressure to the stump before the bone has fully healed [13]. For the permanent prosthesis, we prefer to use an inside tube made from hardened foam, which is made form-fitting using a plaster mould and encompasses the entire lower leg. The prosthesis itself is made from a hard cover and a prosthetic foot is attached to the distal end. This enables the patient to wear normal shoes. From a biomechanical perspective, shear force as little as possible should be applied to the distal area of the amputated stump, especially in the first period of mobility, to prevent a dislocation of the calcaneus. The prosthetic shoe should be placed lightly lateral to the midline at the coronary level to enhance lateral stability. Permanent prosthetic care can be implemented upon removal of the external fixator, usually after the third postoperative month. Our experience shows that the mobilisation of the patient usually occurs without problems. Even older patients quickly learn to walk with the prosthesis and rate the level of disability in everyday life as low [14]. 

## 3. Materials and Methods

Between the years 2000 and 2006, 27 patients were operated on in the Department of Plastic Surgery in the Martin Luther Hospital in Berlin. 20 of the 27 total patients amputated using the Pirogow method were included in the study 12 months after the operation and were evaluated using the Ankle Score from Taniguchi et al. [15]. Five of the seven patients included in the study had to undergo lower leg amputations due to postoperative complications (wound healing dysfunctions), and two patients could no longer be reached at the time of the followup 12 months after the amputation. The Ankle Score is based on the Ankle and Hindfoot Score from the American Orthopaedic Foot and Ankle Society (AOFAS) with regard to loss of ankle function and is more heavily weighted for functional and radiological criteria based on Taniguchi (Table 2). Using a point system, the criteria pain, functional and radiological assessment, difference in leg length, and mobility without a prosthesis were rated on a scale from 0 to 100. The functional assessment is divided into the categories walking range, limping, the ability to go up and down stairs, the ability to stand on one leg, and the ability to sit cross-legged. The radiological points are attributed based on the level of bone atrophy and the assessment of the plantar soft tissue layer. With regard to difference in leg length, the points are given based on length in cm, mobility without a prosthesis and based on the walking range. The maximum value possible is 100 points, and a value above 80 points is rated as an “excellent” result, a value between 60 and 79 points as a “good” result, between 40 and 59 points as an “satisfactory” result, and less than 40 points as a “unsatisfactory” result (Table 2). The Ankle Score Questionnaire was given to 10 patients 12 months (plus or minus 2 weeks) after undergoing the modified amputation based on the Pirogow method. The control group, consisting of 10 patients who underwent the original amputation, was given the same questionnaire in the same time frame (12-month follow-up). 

## 4. Results

The average age of patients who underwent the modified amputation based on the Pirogow method (modified and original) was 58.6 years, with the youngest patient being 32 years old and the oldest patient 76 years old. 15 patients were males (75%), and 5 were females (25%). In 6 of the cases, the right extremity was operated on (60%), in 4 cases the left extremity (40%). 14 of all patients (70%) were operated on based on the indication of diabetes mellitus with necrosis/gangrene in the front or middle foot area causing an amputation to be necessary. In 2 cases (10%), the indication of an amputation based on the Pirogow method was osteomyelitis. One patient (5%) exhibited osteosarcoma in the forefoot, and one patient (5%) was amputated following the Pirogow method due to a severe case of PAOD (IV°) with an ulcer stemming from a forefoot amputation which had been previously carried out. 

The analysis of the Ankle Scores (ASs) from the group of patients that were successfully amputated using the modified Pirogow method resulted in the following point totals (Table 3): 20% report an excellent and 50% a good result (together 70%) compared to 30% claiming an satisfactory and unsatisfactory result. If we examine the individual subcategories of the total AS, we see the following results with regard to the level of pain experienced after being modified amputated: 5 patients (50%) indicated in the 12-month follow-up experiencing no pain (pain free), 4 patients (40%) indicated experiencing light or moderate pain, and one patient (10%) indicated experiencing a high level of pain. If one defines a good result regarding the functional result with a point total >20 with a maximum point total of 40, then 6 patients (60%) indicated a positive functional result 12 months postoperatively. Radiological criteria led to a minimum point total of 0 points; at 12 months after the Pirogow amputation, a trabecula atrophy or bone necrosis persisted. One patient (10%) exhibited this condition. With regard to the difference in leg length, our examination led to the following results: one patient had no difference (10%), 7 patients exhibited a difference <2 cm (70%), and 3 patients exhibited a difference >2 cm (30%). With regard to the level of mobility without a prosthesis, two patients indicated complete mobility (20%), 6 patients (60%) indicated >10 meters, and only 2 patients (20%) indicated being able to move less than 10 meters. 

In the control group, consisting of patients having undergone the original amputation method described by Pirogow, the analysis of the Ankle Scores (AS) resulted in the following point totals (Table 4): 20% report an excellent and 20% a good result (together 40%), whereas 60% report a satisfactory or unsatisfactory result. If we examine the individual subcategories of the total AS, we see the following results in regard to the level of pain experienced after being amputated using the original method: 4 patients (40%) indicated in the 12-month follow-up experiencing no pain (pain free), 2 patients (20%) indicated experiencing light pain, one patient (10%) moderate pain, and one patient (10%) indicated experiencing a high level of pain. If one defines a good functional result with a point total >20 with a maximum point total of 40, then 6 patients (60%) indicated a positive functional result 12 months postoperatively, comparable to the modified method. Differences are shown along radiological criteria in the control group: two patients (20%) exhibited an atrophy of bone trabeculae which was radiologically traceable, one patient a bone necrosis (10%). With regard to the difference in leg length, our examination revealed a major drawback to the original amputation method, namely, that all patients exhibited a difference >2 cm resulting in an invariable point total of 0 points. Patients having undergone the original Pirogow's amputation indicated a decreased level of mobility without a prosthesis <10 m of 4 patients (40%). 

## 5. Discussion

The problems stemming from lower leg amputations induced N.I. Pirogow to describe a new method of amputation in his original article from 1854. When examining the medical situation from a historical perspective, one can undoubtedly state that the level of prosthetic care towards the end of the 19th century was in no way comparable to the options available today. Simple wooden prostheses or the complete absence of prosthetic care of any kind were the main forms of rehabilitation available at the time of the Crimean War [1, 3]. Using the method of amputation at ankle level, Pirogow successfully enabled patients to stay mobile without a prosthesis. Neither the original article nor further literary research reveals any information regarding the level of mobility as far as distance is concerned. If one examines the information gathered from the patients of the modified Pirogow operation method participating in our study, 20% indicated unrestricted mobility. 40% of the patients indicated a mobility distance of greater than 10 meters. In this case, one could criticise the assessment criteria of the Ankle Score, seeing as how it is unclear if a patient who can walk 3 km without a prosthesis after undergoing an amputation based on the modificated Pirogow method assesses this as unrestricted, complete mobility, or merely greater than 10 meters. Adding the option of unrestricted mobility (20%) with the option of mobility greater than 10 meters (40%) shows that 60% of the patients could walk more than 10 meters without a prosthesis, which is an important advantage in particular for mobility in and around the home because although prosthetic care today has been much improved, it is sometimes still necessary to be mobile without a prosthesis, for instance, when going to the bathroom in the middle of the night. In the control group of the original operated patients, 40% indicated a mobility without prothesis <10 m. The patients in both groups were explicitly informed that information given when answering the Ankle Score Questionnaire should only be given regarding mobility without any type of walking assistance (i.e., walkers, crutches, etc.). 

A further advantage of the modified Pirogow amputation method is the fixation of the calcaneus carried out using a Kirschner wire and a 3/0 spongiosa tension screw. Additionally, the calcaneus was anchored by drilling Steinmann nails cranial and anterior to the calcaneal spur to allow the use of an external fixator. These leads to a secure fracture healing. According to our results, radiological complications could be seen in 30% of the patients of the control group in comparison to 10% in the modified group. Because of the fact that we rotated the calcaneus as an additional modification, we saw a decreased difference in the postoperative leg length. The longer length of the lower extremity after undergoing the modified amputation based on the Pirogow method must be seen as another advantage of this method. Taniguchi et al. describe a significantly reduced level of physical strain placed on patients amputated with a difference in length <2 cm [7]. If one examines the results of the patient survey conducted with regard to the final results, one sees different results for the modified method of Pirogow's amputation and original method. 70% of the patients undergoing the modificated Pirogow's amputation indicated an excellent or good result, whereas 40% of the patients indicated these results after the original amputation procedure. In addition, the assessment of pain felt after the different amputation methods showed different results: 50% of those patients amputated by the modified procedure indicated being completely pain free (40% light or moderate pain), while 40% of those original amputated indicated being completely pain free (30% light or moderate pain). The functional results in both patient groups were similar; however, it must be noted that the functional results with regard to mobility were indicated with the help of a prosthesis. The rate of complications must be examined critically. A direct comparison between the two groups regarding the respective complication rate cannot be made, due to the fact that the group's sample size was adjusted with respect to the common criterion “postoperative complications.” The rate of complications following a Pirogow's amputation can be reduced by strictly restricting patients based on indications [16]. For this reason, the authors especially recommend the modified Pirogow method for patients without vascular damage and particularly for patients with traumatic forefoot lesions where preservation of the forefoot through free or vascularised microsurgical flap transplantation is not possible. However, if the modified Pirogow's amputation is carried out successfully, it would present a significant increase in the patient's quality of life due to decreased radiological complications with pain relief, the increased mobility without a prosthesis, and the minimal difference in leg length. 

## 6. Summary

The modified method compared to the original method is characterized by rotating the calcaneus. The calcaneus is relocated vertically 60°–90° to the plantar level approx. 3 cm proximal to the calcaneocuboid joint surface. To fix the calcaneus to the tibia, osteosynthesis can be carried out using a Kirschner wire and a 3/0 spongiosa tension screw. For additional modification, the calcaneus is anchored by drilling Steinmann nails cranial and anterior to the calcaneal spur to allow the use of an external fixator. Ten patients who underwent an amputation based on this modified Pirogow method were surveyed 12 months postoperatively using the Ankle Score (AS) patient questionnaire from Taniguchi et al., based on the Ankle and Hindfoot Score from the American Orthopedic Foot and Ankle Society (AOFAS). Using a point system (0–100 points), the criteria pain, functional and radiological assessment, difference in leg length, and mobility without a prosthesis were recorded and evaluated. An identical questionnaire was given to the control group after the same 12-month time period postoperative; this group consisted of ten patients who had undergone the original Pirogow's amputation method. The modified Pirogow's amputation presents a valuable alternative to the original amputation method for patients with the corresponding indications. The benefits are found in the significantly reduced pain, difference in reduced radiological complications, the increase in mobility without a prosthesis, and the reduction of postoperative difference in leg length.

## Figures and Tables

**Table 1:** Indications and contraindications for an amputation following the Pirogow method [6, 7].

Indications	Contraindications
Congenital foot deformity	Severe circulatory problems
Forefoot and metatarsal damage due to soft tissue or bone tumors	Open lesions or traumatic damage in the area of the heel and calcaneus
Osteomyelitis	Severe immune suppression, cachexy
Burns and frostbite	Acute and chronic infections of the ankle and hindfoot
Persistent ulcerating soft tissue defects stemming from cardiovascular diseases	Low prospects regarding the expected walking ability
Forefoot and metatarsal damage through trauma	Haemodynamically relevant stenosis or closure of the anterior tibialis

**Table 2:** Modified Ankle and Hindfoot Score (AOFAS) with consideration given to the lack of ankle function and more weight given to functional and radiological criteria based on Taniguchi (Ankle Score) [15].

Criteria	Assessment (point total)			
Pain	Pain free (40)	Light (30)	Moderate (20)	Strong (10)
Functional criteria				
Walking distance	Unrestricted (20)	2 km (15)	500 m–2 km (10)	At home (5)
Limping		No Limping (4)	Moderate (2)	Unable to walk (0)
Going up stairs		Unrestricted (4)	Holding the railing (2)	N.m. (0)
Going down stairs		Unrestricted (4)	Holding the railing (2)	N.m. (0)
Standing on one leg		Unrestricted (4)	With support (2)	N.m. (0)
Cross-legged		Unrestricted (4)	With support (2)	N.m. (0)
Radiological criteria				
Bone atrophy		None (5)	Trabecula atrophy (3)	Necrosis (0)
Plantar soft tissue		>2 cm (5)	1-2 cm (3)	<1 cm (0)
Leg length difference		None (5)	<2 cm (3)	>2 cm (0)
Mobility w/out a prosthesis		Unrestricted (5)	>10 m (3)	<10 m (0)

Total points (100)	Excellent (>80)	Good (60–79)	Satisfactory (40–59)	Unsatisfactory (<40)

**Table 3:** The analysis of the Ankle Scores (AS) from the group of patients using the modified method based on Pirogow.

Point total	Result	Number of patients	Percent
>80	Excellent	2	20%
60–79	Good	5	50%
40–59	Satisfactory	3	30%
<40	Unsatisfactory	1	10%

**Table 4:** The analysis of the Ankle Scores (AS) from the group of patients that underwent the original Pirogow's amputation.

Point total	Result	Number of patients	Percent
>80	Excellent	2	20%
60–79	Good	2	20%
40–59	Satisfactory	3	30%
<40	Unsatisfactory	3	30%
